# Inter- and Intrarater Agreement of Spot Sign and Noncontrast CT Markers for Early Intracerebral Hemorrhage Expansion

**DOI:** 10.3390/jcm9041020

**Published:** 2020-04-04

**Authors:** Jawed Nawabi, Sarah Elsayed, Helge Kniep, Peter Sporns, Frieder Schlunk, Rosalie McDonough, Gabriel Broocks, Lasse Dührsen, Gerhard Schön, Thomalla Götz, Jens Fiehler, Uta Hanning

**Affiliations:** 1 Department of Radiology, Charité—Universitätsmedizin Berlin, Corporate Member of Freie Universität Berlin, Humboldt-Universität zu Berlin, and Berlin Institute of Health, 10117 Berlin, Germany; 2 Department of Diagnostic and Interventional Neuroradiology, University Medical Center Hamburg-Eppendorf, 20251 Hamburg, Germany; s.elsayed@uke.de (S.E.); h.kniep@uke.de (H.K.); peter.sporns@hotmail.de (P.S.); rosevmcd@gmail.com (R.M.); g.broocks@uke.de (G.B.); fiehler@uke.de (J.F.); u.hanning@uke.de (U.H.); 3 Department of Neuroradiology, University Hospital Basel, 4031 Basel, Switzerland; 4 Department of Neuroradiology, Universitätsmedizin Berlin, Corporate Member of Freie Universität Berlin, Humboldt-Universität zu Berlin, and Berlin Institute of Health, 10117 Berlin, Germany; frieder.schlunk@charite.de; 5 Department of Neurosurgery, University Medical Center Hamburg-Eppendorf, 20251 Hamburg, Germany; l.duehrsen@uke.de; 6 Institute of Medical Biometry and Epidemiology, University Medical Center Hamburg-Eppendorf, 20251 Hamburg, Germany; g.schoen@uke.de; 7 Department of Neurology, University Medical Center Hamburg-Eppendorf, 20251 Hamburg, Germany; thomalla@uke.de

**Keywords:** computed tomography, intracranial hemorrhage, hematoma expansion, CT marker, interrater reliability, intrarater reliability

## Abstract

Background: The aim of this study was to assess the inter- and intrarater reliability of noncontrast CT (NCCT) markers [Black Hole Sign (BH), Blend Sign (BS), Island Sign (IS), and Hypodensities (HD)] and Spot Sign (SS) on CTA in patients with spontaneous intracerebral hemorrhage (ICH). Methods: Patients with spontaneous ICH at three German tertiary stroke centers were retrospectively included. Each CT scan was rated for four NCCT markers and SS on CTA by two radiology residents. Raters were blind to all demographic and outcome data. Inter- and intrarater agreement was determined by Cohen’s kappa (κ) coefficient and percentage of agreement. Results: Interrater agreement was excellent in 473 included patients, ranging from 96% to 99%. Interrater κ ranged from 0.85 (95% CI [0.78–0.91]) to 0.97 (95% CI [0.94–0.99]) for NCCT markers and 0.93 (95% CI [0.88–0.98]) for SS, all *p*-values < 0.001. Intrarrater agreement ranged from 96% to 100%, with κ ranging from 0.85 (95% CI [0.78–0.91]) to 1.00 (95% CI [0.10–0.85]) for NCCT markers and 0.96 (95% CI [0.92–1.00]) for SS, all *p*-values < 0.001. Conclusions: NCCT imaging findings and SS on CTA have good-to-excellent inter- and intrarater reliabilities, with the highest agreement for BH and SS.

## 1. Introduction

Intracerebral hemorrhage (ICH) is the most severe form of stroke with a one month morbidity and mortality rates approaching 50% and death or severe disability exceeding 75% [1,2,3,4]. Factors reflecting the dynamic nature of hematoma evolution are particularly important for clinical outcome [5,6]. As so, early secondary hematoma expansion due to active extravasation and rebleeding has been reported in 38% of patients after initial imaging on computed tomography (CT) and reported as an independent prognostic factor for poor functional outcome [7,8]. It moreover forms an appealing therapeutic target, as early hematoma expansion is potentially modifiable in the acute onset time frame [9,10]. Therefore, identification of patients with ICH and a potential risk of hematoma expansion is crucial for triage of patients and prediction of the functional outcome [1]. To this end, several imaging markers on non-contrast computed tomography (NCCT) have been described to be predictive of hematoma expansion and poor functional outcome [6]. These imaging characteristics include the blend sign (BS) [6,11], the black hole sign (BHS) [6,12,13], the island sign (IS) [6,14], and the more general appearance of hypodensities (HD) [6,15]. In addition, a contrast extravasation on computed tomography angiogram (CTA) within an ICH is referred as the established spot sign [16,17]. Some of the imaging characteristics present each with overlapping definitions and criteria, such as BHS and HD [6]. The issue of error in radiology has been recognized for many years [18]. On the part of radiologists, the “lack of knowledge” and “under reading specific causes” contribute amongst others to the specific causes of error [18]. Nonetheless, there is limited data concerning the formal and independent assessment of the reliability of the above-mentioned hematoma markers [6]. According to a recently published study from Dowlatshahi et al., five NCCT markers were analyzed for inter- and intrarater reliability [19]. Interrater and intrarater reliabilities were good-to-excellent, albeit based on a relatively small patient cohort (n = 40) [19]. In addition, SS on CTA has not been analyzed by Dowlatshahi et al. [19], despite contributing individually the most for not only hematoma expansion but also outcome prediction in patients with ICH when compared to five commonly used NCCT markers [1]. Wada et al. were the first to report the SS on CTA with an excellent interrater reliability, however the number of patients was again relatively small (n = 39) and secondly intrarater reliability was not assessed [20]. We hypothesized that the commonly used NCCT markers and SS on CTA had a high level of inter- and interrater reliability. To test and evaluate this hypothesis, the interrater and intrarater reliability of four commonly reported NCCT markers and the established SS on CTA of hematoma expansion was assessed in a large multicenter cohort.

## 2. Materials and Methods

### 2.1. Study Population

We retrospectively studied the databases of three German tertiary stroke centers for patients with spontaneous ICH aged >18 years between January 2016 and December 2018. (University Hospital of Muenster, University Hospital Hamburg-Eppendorf and Charité University Hospital Berlin).

As inclusion criteria, we defined (1) primary spontaneous ICH confirmed on NCCT confirmed by a senior physician or fellow radiologist with extensive experience in stroke imaging and (2) NCCT and CTA performed on admission within 6 h after symptom onset. Primary spontaneous ICH were included despite severity and size, except for being accompanied by sub- or epidural and subarachnoid hemorrhage. Both databases included patients with anticoagulant treatment, but excluded patients with sub- or epidural hematoma and subarachnoid hemorrhage, and with secondary causes of ICH such as head trauma, brain tumor, vascular malformation, primary intraventricular hemorrhage, or secondary ICH from hemorrhagic transformation of ischemic infarction. Additionally, we obtained vascular risk factors (hypertension and diabetes mellitus) and surgery procedures (craniectomy) from patients’ clinical records. This multicenter retrospective study was approved by the ethics committee (Ethik-Kommission der Ärztekammer Hamburg [WF-054/19], Ethik-Komission der Charité Berlin [EA4/011/20] and Ethik-Komission der Uniklinik Münster [2017-233-f-S]) and written informed consent was waived by the institutional review boards. All study protocols and procedures were conducted in accordance with the Declaration of Helsinki. Due to the retrospective nature of the study, patient consent was not needed.

### 2.2. Image Acquisitions

The CT scans were performed using standard clinical parameters with axial 5 mm section thickness. In detail the employed imaging protocols were the following:

CT scans at the University Hospital Hamburg-Eppendorf were performed on a 256 slice scanner (Philips iCT 256. Amsterdam, Netherlands) with the following imaging parameters: NCCT with 120 kV, 280–320 mA, 5.0 mm slice reconstruction; CTA: 100–120 kV, 260–300 mA, 1.0 mm slice reconstruction, 5 mm MIP reconstruction with 1 mm increment, 0.6-mm collimation, 0.8 pitch, H20f soft kernel, 80 mL highly iodinated contrast medium, and 50 mL NaCl flush at 4 mL/second; scan started 6 s after bolus tracking at the level of the ascending aorta.

CT scans at the Charité University Hospital were performed on a 80 slice scanner (Toshiba Aquilion Prime. Tokio, Japan) with the following imaging parameters: NCCT with 120 kV, 300 mA, 5.0 mm slice reconstruction; CTA: 100–120 kV, dosis-modulated between 260–300 mA, 1.0 mm slice reconstruction, 5 mm MIP reconstruction with 1 mm increment, 0.5-mm collimation, 0.64 pitch, separate reconstruction kernels (brain, FC21; bone, FC30) at the same thickness (1 and 5 mm gapless), 60 mL highly iodinated contrast medium, and 30 mL NaCl flush at 4 mL/second; scan started 6 s after bolus tracking at the level of the ascending aorta.

CT scans at the Muenster Universtiy Hospital were performed on a 2 × 128 slice scanner (Siemens SOMATOM Definition Flash. Erlangen, Germany) with the following imaging parameters: NCCT with 120 kV, 280 mA, 5.0 mm slice reconstruction; CTA: 100–120 kV, between 260–300 mA, 1.0 mm slice reconstruction, 5 mm MIP reconstruction with 1 mm increment, 0.5-mm collimation, 0.8 pitch, H20f soft kernel, 60 mL highly iodinated contrast medium, and 30 mL NaCl flush at 4 mL/second; scan started 6 s after bolus tracking at the level of the ascending aorta.

### 2.3. Image Analysis

The location of the hematoma and presence of intraventricular hemorrhage was assessed and documented. The hemorrhage locations were classified as deep (basal ganglia and thalamic), lobar, or within the brain stem and pons, or cerebellum.

Two raters experienced in stroke imaging independently reviewed images in a random order, blind to all demographic and outcome data and were not involved in the clinical care of assessment of the enrolled patients. They had corresponding experience in neuroradiology and stroke imaging with three years (J.N.) and four years (S.E.) of experience. Images were randomized and presented again to both raters one month later for a second reading, so as to minimize recall of the patients’ follow-up scans.

Raters analyzed axial NCCT images and subsequently the corresponding CTA to determine the presence of the following markers: BS, BHS, IS, HD, and SS were rated in due consideration of the proposed consensus standard of Morotti et al. [6]: In brief, BS was defined as a hypoattenuating area adjacent to a hyperattenuating area of the hematoma, with a clear separation between them at a density difference of at least 18 Hounsfield Units (HU) [11,21]. The BHS consists of a relatively hypodense area which is encapsulated within a hyperdense area and which is not connected with the adjacent brain tissue [12,13]. The relatively hypodense area has an identifiable border and a difference of at least 28 HU between the two density regions [12,21]. The IS consists of at least 3 scattered small hematomas all separate from the main hematoma or at least 4 small hematomas some or all of which may connect with the main hematoma [14]. The imaging sign HD was defined as any hypodense region strictly encapsulated within the hemorrhage with any shape, size, and density which does not require a density measurement [6,15,22]. The spot sign on CTA was well-acknowledged as the foci of enhancement within the intracranial hematoma, detected on CTA source images [23,24].

### 2.4. Statistical Analysis

Level of agreement was calculated as the number of agreements divided by the total number of readings. Interrater and intrarater agreement was calculated and expressed as percentage of agreement and Cohen’s κ statistic with stratified kappa with 95% upper and lower confidence intervals (CI) [25]. The intrarater estimates for percentage agreement were calculated as the percentage of pairs of readings (first reading or second reading) that agreed over all pairs for all raters. The interrater estimates were calculated as the percentage of pairs of readings from pairs of the two raters that were in agreement. For intrarater agreement, kappa calculations were stratified by rater, whereas for interrater agreement, Kappa calculations were stratified by each combination of reading (reading 1 or reading 2) from pairs of the two raters. Analyses were performed using the statistical software package SPSS version 25^®^ (IBM Corporation, Armonk NY) and and R Statistics^®^ Version 3.5.1 (R Core Team. R: A Language and Environment for Statistical Computing. R Foundation for Statistical Computing. Vienna, Austria, 2018).

## 3. Results

The 473 patients had a median age of 69 years (IQR: 67.9–70.6) with 252 female patients (53.3%). A total of 326 (69.1%) patients suffered from arterial hypertension and 65 (13.8%) from diabetes mellitus. Bleeding locations were most frequently located in the basal ganglia and thalamus (deep) in 206 (43.6%) and lobar in 210 (44.4%) patients. Infratentorial bleeding locations consisted of 40 (8.5%) cerebellar and 17 (3.6%) brain stem and pons ICH. A total of 248 patients had intraventricular extension (52.5%). A total of 110 (23.3%) supratentorial and 5 (1.1%) infratentorial craniectomy procedures were performed. A clinical outcome above three on a modified Rankin Scale (mRS) was observed in 326 (68.9%) cases) (Table 1).

**Figure 1:**
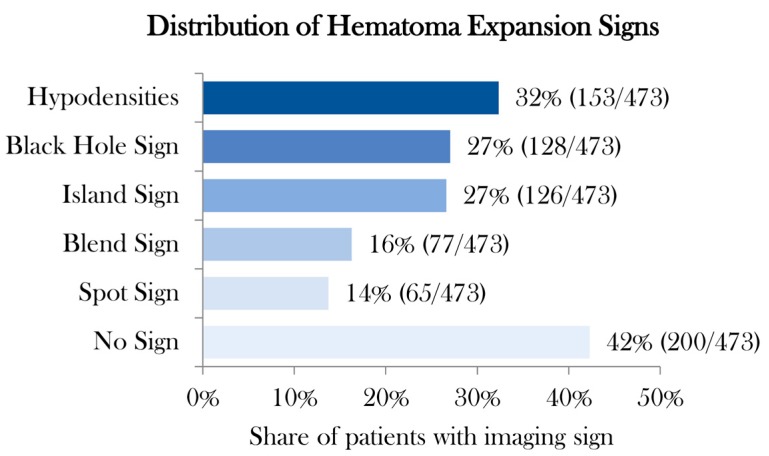
Distribution of hematoma expansion sign. Legend: Distribution in percentage and absolute numbers of four noncontrast computed tomography imaging signs and spot sign on computed tomography angiography.

**Figure 2:**
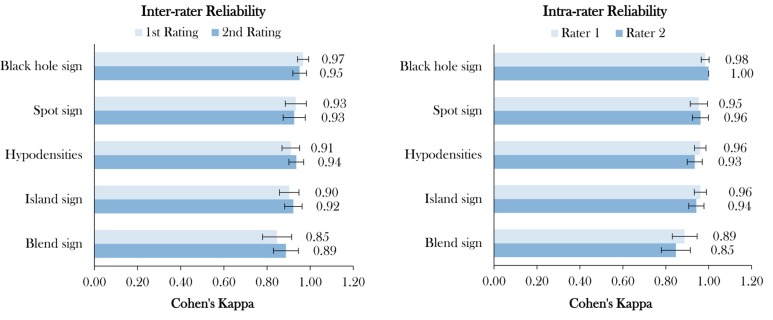
Inter- and intrarater reliability. Legend: Inter- and intrarater agreement of four noncontrast computed tomography imaging signs and spot sign on computed tomography angiography specified with stratified kappa with 95% confidence interval (CI) and listed in descending order; all *p*-values < 0.001.

**Figure 3:**
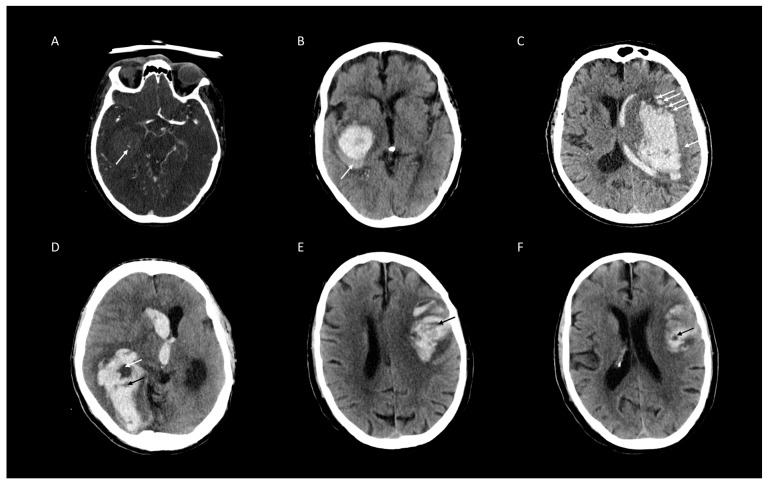
Representative examples of agreed ratings of four non-contrast computed tomographic (NCCT) markers and Spot Sign (SS) on CT-angiography (CTA) for intracerebral hemorrhage expansion. (**A**) SS on CTA (white arrow). (**B**) Blend Sign (white arrow). (**C**) Island sign (all white arrows). (**D**) Black Hole Sign (white arrow), Hypodensities (black arrow). (**E**) Hypodensities (black arrow). (**F**) Black Hole Sign (white arrow).

**Figure 4:**
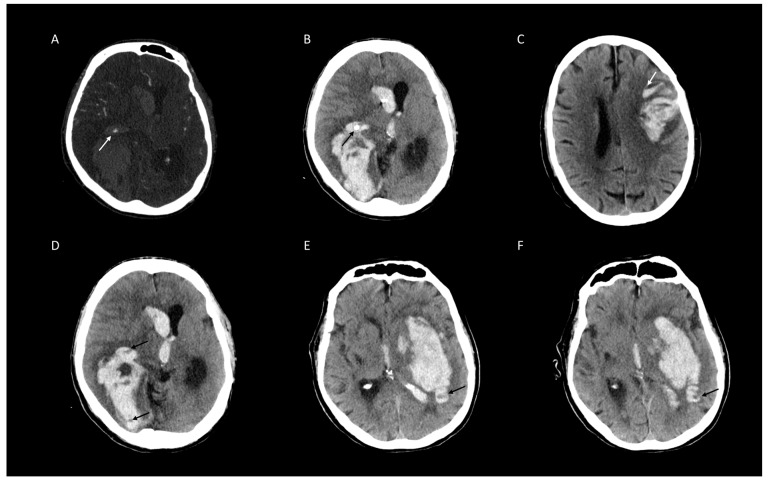
Representative examples of disagreed ratings of four non-contrast computed tomographic (NCCT) markers and Spot Sign (SS) on CT-angiography (CTA) for intracerebral hemorrhage expansion. (**A**) SS on CTA (white arrow) mistaken for intraventricular plexus calcification (black arrow) (**B**). (**C**) Blend sign (white arrows) mistaken for Fluid Sign1. (**D**) Swirl Sign mistaken for Hypodensities (black arrow). (**E**) Hypodensities (black arrow) mistaken for Swirl Sign (**F**) [6].

Of 473 patients with spontaneous ICH, 179 (30%) presented with HD, 125 (26%) with BH, 114 (25%) with IS, 76 (15%) with BS, and 75 (12%) with SS (Figure 1). Distribution of poor clinical outcome (mRS > 3) [26] was higher in patients with the presence of NCCT markers and SS on CTA (Table 2). The distribution of BS, HD, and SS increases rapidly with a coappearance of signs above three. Whereas in coappearance of two to three signs, distribution is predominated by BHS and IS (Table 3). Overall, interrater agreement was excellent for BH, BS, IS, HD and SS in the first (98.7%, 96%, 96.4%, 96.2%, and 98.5%, respectively) and also the second rating (98.1%, 97%, 97%, 97.3%, and 98.3%, respectively). Interrater stratified kappa was the best for BH and SS, and the lowest for IS in the first rating (0.97 (95% CI [0.94–0.99]), 0.93 (95% CI [0.88–0.98]), and 0.90 (95% CI [0.86–0.95]), all *p*-values < 0.001). In the second rating, interrater stratified kappa was the second best for BH and SS, and also the best for HD (0.93 (95% CI [0.92–0.98]), 0.92 (95% CI [0.87–0.98]), and 0.95 (95% CI [0.90–0.97]), all *p*-values < 0.001) (Figure 2 and Table 4). Intrarater agreement and stratified kappa followed a similar pattern with excellent levels of agreement and stratified kappa among both raters with the highest for BH (Rater 1: 100%, 1.0 (95% CI [1.0–1.0]) and Rater 2: 99.4%, 0.98 (95% CI [0.97–1.0]) and SS (Rater 1: 99.2%, 0.96 (95% CI [0.92–1.0]) and Rater 2: 98.9%, 0.95 (95% CI [0.91–0.99]), all *p*-values < 0.001) (Figure 2 and Table 5). Representative images of agreement and disagreement for all four NCCT markers and SS on CTA are illustrated in Figure 3 and Figure 4.

## 4. Discussion

Hematoma expansion is a therapeutic target of clinical interventions and a potentially modifiable predictor of clinical outcome [8,27,28]. There is growing evidence that different imaging markers in NCCT and SS in CTA predict early hematoma expansion and therefore offer great additional value [1,21]. The main finding of our study is that four commonly used NCCT imaging marker and SS on CTA have excellent interrater and intrarater reliabilities. Prevalence of all signs in our study was comparable to other studies [1].

To our knowledge, this is the first study incorporating analysis of interrater and intrarater agreement of markers on NCCT and SS on CTA in a large multicenter cohort without controlling for factors such as image acquisition parameters or scanner type. A recent, large, independent patient data meta-analysis suggested that prediction of ICH expansion was improved by the addition of information on SS from CTA, alongside time from symptom onset, baseline hematoma volume, and antithrombotic medication [27]. According to the recent study of Sporns et al., SS on CTA had a higher sensitivity for clinical outcome prediction than the NCCT imaging markers (BS, BH, IS, HD) alone, and hence recommendable to be acquired collectively [1]. A recently published study by Dowlatshahi et al. analyzed the interrater and intrarater reliability of NCCT imaging markers for hematoma expansion, but SS on CTA was not being assessed [19] which this study has taken this into consideration. Implementing rater assessments of two radiology residents offers complementary yet distinctive information to the results by Dowlatshahi et al., considering that not only neurologists but synergistically also radiology residents are generally the first to analyze the CT scans.

The large patient population suggests a high reliability and importance of these imaging parameters for outcome prediction in patients with ICH. Further, there is considerable interest in hematoma expansion prediction scores that incorporate NCCT markers [15]. Taking into account that SS has a higher sensitivity for outcome prediction than BS and hypodensity alone, both NCCT and CTA should be acquired if possible [1]. In accordance with the evaluation of reliabilities in our study it would be conceivable to improve such prediction scores by adding SS on CTA. Scores with NCCT markers as the BAT Score still have their ‘raison d’être in a setting where CTA is not readily available or with strong contraindications for contrast application (distinct allergy, far progressed renal dysfunction), sole acquisition of noncontrast CT and evaluation of BS and BHS is a valuable option for detecting hematoma growth associated with poor outcome—as the NCCT imaging markers of BS and BHS are strongly correlated with the established SS. In line with this, our results indicate a strong increase in the distribution of markers coappearance above 3 imaging signs of BS, SS, and HD. Whereas coappearance in one to three signs is predominated by BHS and island sign.

In conclusion, this study had several limitations. Firstly, the level of experience was not being evaluated. In this study, the reading time was not taken under consideration as image interpretation in an emergency setting is much more complicated, rushed, and confusing compared to elective scans. Further prospective studies are required to validate the use of NCCT and CTA markers in clinical practice and should take into consideration both the level of experience and reading time.

In conclusion, the high interrater and intrarater reliability suggests that all NCCT signs and SS are easy to use, thereby supporting their use in emerging scores and development and validation of machine learning tools to predict hematoma expansion, or either within randomized clinical or therapeutic trials targeting hematoma expansion [6,15,16,29].

## Figures and Tables

**Table 1:** Comparison of baseline demographic and radiological characteristics.

Baseline Clinical and Imaging Characteristics	All (n= 473)
**Clinical Characteristics**	
Age at admission [years], median (IQR)	69.25 (67.9–70.6)
Female, n (%)	252 (53.3)
Hypertension, n (%)	326 (69.1)
Diabetes mellitus, n (%)	65 (13.8)
**Imaging Characteristics**	
Bleeding location, n (%)	
• Deep	206 (43.6)
• Lobar	210 (44.4)
• Brain Stem, Pons	17 (3.6)
• Cerebellum	40 (8.5)
Intraventricular hemorrhage, n (%)	248 (52.5)
**Surgery procedures**	
Supratentorial Craniectomy, n (%)	110 (23.3)
Infratentorial Craniectomy, n (%)	5 (1.1)
**Clinical outcome, n (%)**	
mRS ≤ 3	147 (31.1)
mRS > 3	326 (68.9)

Legend: % indicates percentage; IQR indicates interquartile range; n indicates absolute number.

**Table 2:** Distribution of modified Rankin Scale at discharge according to the presence of imaging markers for early hematoma expansion in patients with spontaneous intracerebral hemorrhage.

Imaging Signs for ICH Expansion	Presence	mRS [median]	mRS > 3 [%]
All NCCT Signs and Spot Sign on CTA	no	3.3	47%
	yes	4.8	85%
Black Hole Sign	no	3.8	59%
	yes	5.4	95%
Blend Sign	no	4.1	65%
	yes	4.9	92%
Hypodensities	no	4.1	65%
	yes	4.5	77%
Island Sign	no	3.9	61%
	yes	5.2	92%
Spot Sign	no	4.1	65%
	yes	5.1	95%

Legend: ICH indicates intracerebral hemorrhage; mRS modified Rankin Scale; NCCT noncontrast computed tomography imaging signs and spot sign on computed tomography angiography.

**Table 3:** Distribution of simultaneous appearance of imaging markers for early hematoma expansion in patients with spontaneous intracerebral hemorrhage.

Number of Imaging Signs	Number of Patients	mRS [median]	mRS > 3 [%]	Black Hole	Blend Sign	Island Sign	Hypodensities	Spot Sign
0	185	3	45%	0%	0%	0%	0%	0%
1	129	4	74%	27%	15%	11%	39%	9%
2	92	5	89%	51%	20%	63%	51%	15%
3	42	6	98%	62%	40%	79%	74%	45%
4	15	5	87%	67%	87%	73%	100%	73%
5	10	5	100%	100%	100%	100%	100%	100%
Total	473	4	69%	27%	16%	27%	32%	14%

Legend: Distribution of simultaneous appearance of imaging signs and clinical outcome by number of simultaneously seen imaging signs.

**Table 4:** Interrater agreement of two raters stratified across two readings.

Rater 1	Rater 2	Level Agreement	Cohen’s kappa *	95% Lower CI	95% Upper CI	z-Statistic	*p*-Value
1st Rating							
Black Hole	Black Hole	98.7%	0.97	0.94	0.99	21.0	<0.001
Spot Sign	Spot Sign	98.5%	0.93	0.88	0.98	20.3	<0.001
Hypodensities	Hypodensities	96.2%	0.91	0.87	0.95	19.8	<0.001
Island Sign	Island Sign	96.4%	0.90	0.86	0.95	19.7	<0.001
Blend Sign	Blend Sign	96.0%	0.85	0.78	0.91	18.4	<0.001
2nd Rating							
Black Hole	Black Hole	98.1%	0.95	0.92	0.98	20.7	<0.001
Spot Sign	Spot Sign	98.3%	0.93	0.87	0.98	20.2	<0.001
Hypodensities	Hypodensities	97.3%	0.94	0.90	0.97	20.4	<0.001
Island Sign	Island Sign	97.0%	0.92	0.88	0.96	20.1	<0.001
Blend Sign	Blend Sign	97.0%	0.89	0.83	0.95	19.3	<0.001

Legend: Interrater agreement of four noncontrast computed tomography imaging signs and spot sign on computed tomography angiography specified with percentage agreement and stratified kappa with 95% confidence interval (CI). * stratified Cohen’s kappa across two raters in two ratings.

**Table 5:** Intrarater agreement of two readings stratified across two raters.

1st Rating	2nd Rating	Level of Agreement	Cohen’s kappa *	95% Lower CI	95% Upper CI	z-Statistic	*p*-Value
Rater 1							
Black Hole	Black Hole	100%	1.00	1.00	1.00	21.75	<0.001
Island Sign	Island Sign	97.9%	0.94	0.91	0.98	20.50	<0.001
Hypodensities	Hypodensities	97.3%	0.93	0.90	0.97	20.34	<0.001
Spot Sign	Spot Sign	99.2%	0.96	0.92	1.00	20.92	<0.001
Blend Sign	Blend Sign	96.0%	0.85	0.78	0.91	18.44	<0.001
Rater 2							
Black Hole	Black Hole	99.4%	0.98	0.97	1.00	21.40	<0.001
Island Sign	Island Sign	98.5%	0.96	0.93	0.99	20.93	<0.001
Hypodensities	Hypodensities	98.3%	0.96	0.93	0.99	20.91	<0.001
Spot Sign	Spot Sign	98.9%	0.95	0.91	0.99	20.77	<0.001
Blend Sign	Blend Sign	97.0%	0.89	0.83	0.95	19.33	<0.001

Legend: Intrarater agreement of four noncontrast computed tomography imaging signs and spot sign on computed tomography angiography specified with percentage agreement and stratified kappa with 95% confidence interval (CI). * stratified Cohen’s kappa across one rater in two ratings.
